# A Novel Nonparametric Feature Selection Approach Based on Mutual Information Transfer Network

**DOI:** 10.3390/e24091255

**Published:** 2022-09-07

**Authors:** Kunmei Li, Nasser Fard

**Affiliations:** College of Engineering, Northeastern University, Boston, MA 02115, USA

**Keywords:** feature selection, mutual information, linear programming, maxflow, P-median problem

## Abstract

The filter feature selection algorithm is habitually used as an effective way to reduce the computational cost of data analysis by selecting and implementing only a subset of original features into the study. Mutual information (MI) is a popular measurement adopted to quantify the dependence among features. MI-based greedy forward methods (MIGFMs) have been widely applied to escape from computational complexity and exhaustion of high-dimensional data. However, most MIGFMs are parametric methods that necessitate proper preset parameters and stopping criteria. Improper parameters may lead to ignorance of better results. This paper proposes a novel nonparametric feature selection method based on mutual information and mixed-integer linear programming (MILP). By forming a mutual information network, we transform the feature selection problem into a maximum flow problem, which can be solved with the Gurobi solver in a reasonable time. The proposed method attempts to prevent negligence on obtaining a superior feature subset while keeping the computational cost in an affordable range. Analytical comparison of the proposed method with six feature selection methods reveals significantly better results compared to MIGFMs, considering classification accuracy.

## 1. Introduction

**Figure 1:**
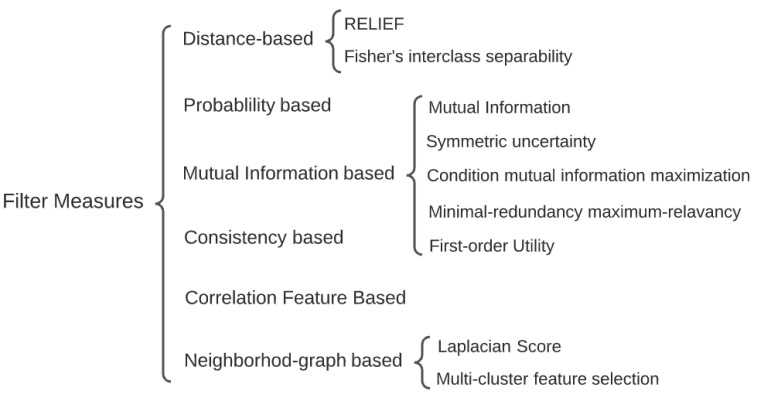
Filter measures summary.

The ‘Curse of Dimensionality’ is first mentioned by Bellman to describe various challenges that occur during data analysis due to increasing dimensionality [1]. Most of those challenges refer to computational complexity, especially the exhaustion of computational storage and time. Multiple types of dimensionality reduction (DR) approaches, such as feature selection (FRS) and feature extraction, have been proposed to overcome computational fatigue. FRS focuses on selecting a proper subset of original features from the data to maximize or minimize predetermined objectives to meet the requirements of various users.For instance, the objective can be to minimize the Akaike Information Criterion (AIC)/the Bayesian Information Criterion (BIC) or to maximize the classification accuracy [2]. FRS has also been extensively applied because of its capability to promote model interpretability. By implementing various classifiers along with machine learning approaches, FRS approaches are grouped into three types: filter, wrapper, and embedded methods. Filter methods refer to the FRS approaches without considering the classifier [3]. Embedded methods are similar to wrapper methods, since they both choose features that optimize the classifier’s performance, except embedded methods have an intrinsic model-building metric that wrapper methods do not have during learning process. Filter methods are more computationally efficient than wrapper and embedded methods, since they do not take classifiers into account [4]. The filter methods are classified into several types based on the measures of relationship between predictors and response (or target), including distance-based, probability-based, MI-based, consistency, and correlation measures [5]. Figure 1 presents a summary of some filter measures.

Compared to other types of filter measures, MI-based measures have a few superiorities. To begin with, MI-based measures can assess the general dependence among random variables without making any assumptions in advance. Furthermore, MI-based measures are slightly affected by the monotone transformation and classifier selection [6]. These advantages allow MI-based measures for broad application in the analysis of various types of problems, including computer-aided diagnosis [7], cyber intrusion detection [8], heart failure recognition [9], and software cost estimation [10].

MI-based greedy forward methods (MIGFMs) are widely used for high-dimensional classification problems to avoid computational complexity and exhaustion. It follows the heuristic of making a greedy choice at each step of searching process based on a given optimal criterion to ensure the optimality of feature selection, which significantly reduces the searching process. For instance, to select *m* features out of *n* possible features, the total number of all the candidate subsets is nm=n!m!(n−m)!, which could be enormous for a relatively large *n*. In the greedy forward methods, features are selected incrementally, and only one feature is selected from the candidate subsets consisting of unselected features at each searching step based on a specified optimal criterion. Consequently, the total number of searches for determining *m* out of *n* features is:

(1) $$n+\left(n-1\right)+\left(n-2\right)+\cdots +\left(n-m+1\right)=\frac{(2n-m+1)m}{2}$$

This is significantly smaller compared to $\frac{n!}{m!(n-m)!}$. More details about MIGFMs are given in Section 2.

Even though greedy forward methods deduct the total number of steps, they may simultaneously lead to negligence of a superior subset. Greedy algorithms typically lack the foresight to select inferior options during an iteration to facilitate subsequent results from the algorithm [11]. Moreover, they encounter challenges in assessing the impact of interactions among features [12]. To overcome the aforementioned limitations and to balance the efficiency and accuracy of MI-based FRS methods, we propose a new nonparametric approach based on MILP in Section 3. We will also compare the performance of the proposed method with several popular MI-based heuristic FRS methods and provide a discussion in Section 4 and Section 5.

## 2. Information Theory and MI-Based Methods

This section briefly introduces the basic concepts of information theory and then presents a few MIGFMs with various optimal criteria.

### 2.1. Information Theory

Information theory is part of probability theory that was initially proposed to measure information transmitted across a noisy channel [13]. It has been broadly applied to diverse fields, such as computer science, social science, economics, and physics [14]. Two parameters, entropy and MI, are used to measure the amount of information. For a random discrete variable $X=\{x_{1},x_{2},x_{3},\ldots ,x_{n}\}$, the average information about *X* is represented by Shannon entropy H(X), where $p_{i}$ denotes the probability of $x_{i}$.

(2) $$H\left(X\right)=-\sum_{i=1}^{n}p_{i}log(p_{i})$$

If there is another random discrete variable *Y*, the amount of information needed to describe *X* given a known *Y* is denoted by conditional entropy H(X|Y):

(3) H(X|Y)=H(X,Y)−H(Y)

where H(X,Y) is the joint entropy measuring the entropy associated with these two variables:

(4) $$H\left(X,Y\right)=-\sum_{x\in X}\sum_{y\in Y}p\left(x,y\right)logp(x,y)$$

The mutual information of *X* and *Y* measures the information about *X* (or *Y*) that can be obtained through *Y* (or *X*) and is defined as:

(5) I(X;Y)=H(X)−H(X|Y)=H(Y)−H(Y|X)

These definitions can be expanded to multivariate cases, which are primarily expressed by the following two equations that involve conditional entropy and conditional mutual information.

(6) $$H\left(X_{1},X_{2},\ldots ,X_{n-1},X_{n}\right)=\sum_{i=1}^{n}H\left(X_{i}|X_{i-1},\ldots ,X_{1}\right)$$

(7) $$\begin{array}{cc} I(X_{1},X_{2},\ldots ,X_{n};Y) & =\sum_{i=1}^{n}I\left(X_{i};Y|X_{i-1},\ldots ,X_{1}\right)\\ \; & =H\left(X_{1},X_{2},\ldots ,X_{n}\right)-H\left(X_{1},X_{2},\ldots ,X_{n}|Y\right)\; \end{array}$$

**Figure 2:**
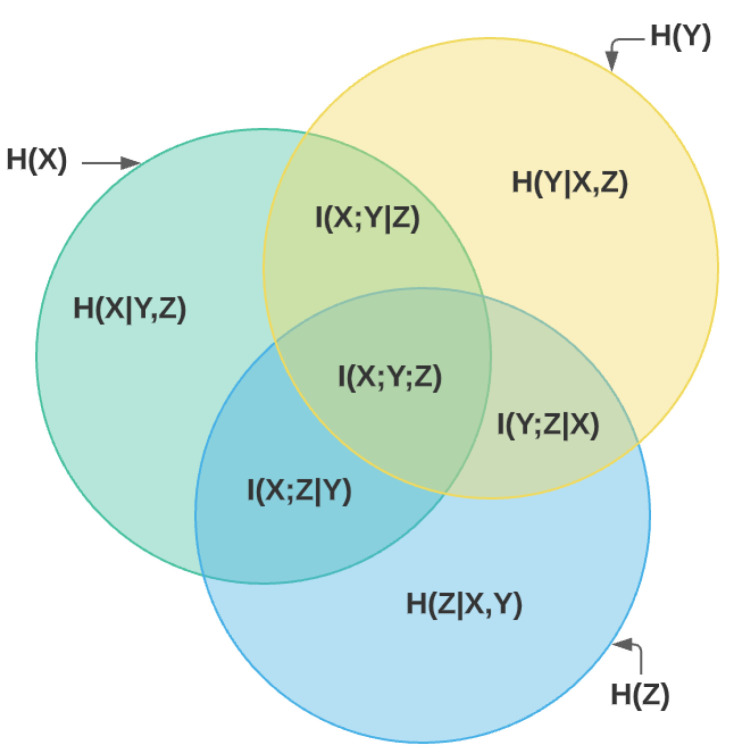
Three-variable MI venn diagram.

Figure 2 presents a Venn diagram that distinctly describes the relationships between entropy and mutual information of three variables [15]. The above concepts are also generalized for the case of continuous variables by replacing partial equations with differential equations. Furthermore, various approximation approaches have been proposed to measure the mutual information between discrete and continuous variables, such as Jensen Shannon divergence (JSD) [16] and kernel density approximation (KDA) [17].

### 2.2. MI-Based FRS Methods

As mentioned above, FRS seeks to obtain a subset of features that provides the optimum performance for a specific objective. For the MI-based methods, the objective could be to maximize the mutual information between a selected predictor and a class variable in a classification task. For example, suppose that there is a data set with *n* features denoted by $\left\{f_{1},f_{2},f_{3},\ldots ,f_{n-1},f_{n}\right\}$ and one class variable denoted by *C*. A selected subset consisting of *m* features, $S^{{}^{\prime }}$, should satisfy:

(8) $$S^{{}^{\prime }}=arg\max_{S}\left\{I\left(C;S\right)\right\}$$

where S denotes all the possible combinations of *m* features. The total number of the S is nm, which can be a huge number for a large *n*, as stated in Section 1. To avoid computational complexity and exhaustion caused by a large *n*, various greedy forward methods focusing on the systematic selection of features have been proposed. Including MI variable selector under uniform information distribution (MIFS-U) [18], information gain feature selection (IGFS) [19], conditional informative feature extraction (CIFE) [20], min-redundancy and max-dependency (MRMD) [21], max-relevance and max-independence (MRI) [22], max-relevance and min-redundancy (mRMR) [23], and spectral relaxation conditional MI (SPEC-CMI) [24]. These MIGFMs are summarized in Algorithm 1.
**Algorithm 1** Summarized Algorithm.1:Initialization: SEL=∅, $\bar{SEL}=\left\{f_{1},f_{2},f_{3},\ldots ,f_{n-1},f_{n}\right\}$, *m* is the number of features to be selected, and 0<m≤n2:**while**|SEL|<m**do**:3:    $f\leftarrow arg\max_{f_{i}\in \bar{SEL}}\left\{I\left(C;f\right)-\beta \sum_{f_{i}\in SEL}g\left(f,f_{i},C\right)\right\}$4:    SEL←SEL+{f}5:    $\bar{SEL}\leftarrow \bar{SEL}-\left\{f\right\}$6:**end while**7:**return**SEL

Where β is a critical scale parameter, $g(f,f_{i},C)$ is a manually set function, SEL is the set of selected features, and the |SEL| denotes the set cardinality of SEL. In each search step, an unselected feature that maximizes the locally optimal function $I\left(C;f\right)-\beta \sum_{f_{i}\in SEL}g\left(f,f_{i},C\right)$ is picked.

Multiple kinds of MI-based FRS methods can be transformed into this algorithm with different β and $g(f,f_{i},C)$ as provided in Table 1.

In addition to the methods indicated in Table 1, the methods with nonlinear combinations of mutual information, such as CMIM [25], ICAP [26], NMIFS [27], and DISR [28] are also adopted in various aspects [29].

When employing greedy forward methods, some concerns need to be specified, such as selecting appropriate parameters such as β and the stopping criterion of the greedy procedure [30]. The improper specification of parameters and stopping criteria may lead to a negligence of obtaining a better subset [31]. To circumvent the disadvantages of the parametric methods, we present a novel method which converts the FRS problem into a mixed-linear optimization model. P-median method is used for the selection of facility location according to an objective function, similarly MILP approaches could be introduced for feature selection. The following section introduces some basic definitions of the P-median model, as it relates to our proposed method.

## 3. P-Median Problem and Proposed Algorithm

In this section, we first present a brief description of the P-median problem before presenting the proposed algorithm.

### 3.1. P-Median Problem

The P-median problem is an imperative type of discrete location problem that focuses on selecting optimal locations for *P* facilities from a set of potential locations to minimize the total delivery cost or distance [32]. The selection of suitable locations for facilities is one of the most critical tasks in almost every sector. For instance, the transportation authority must determine the locations of bus and subway stations; the government needs to locate public services, including hospitals and schools; the delivery services company is required to locate the mail drop boxes. Such strategical problems are known as discrete location problems or facility location problems. P-median problem is an NP-hard problem that takes polynomial time to solve [33]. As the number of variables and constraints involved in this problem grows, the complexity of the P-median problem increases, and when the number of arcs in graphs built from the P-median problem exceeds 360,000, commercial MILP coding becomes difficult [34]. Therefore, instead of seeking a globally optimal result, we set termination criteria such as elapsed time, relative gap tolerance, and absolute gap tolerance to identify a relatively optimum solution that can be recognized within a reasonable amount of time while maintaining acceptable accuracy. Typically, the P-median problem is formulated as following MILP:

(9) $$\begin{array}{ccc} \mathrm{minimize} & \sum_{i\in I}\sum_{j\in J}b_{i}d_{ij}y_{ij}\\ \mathrm{subject}\;\mathrm{to} & \sum_{j\in J}x_{j}=P\\ \; & \sum_{j\in J}y_{ij}=1, & \forall i\\ \; & y_{ij}-x_{j}\leq 0, & \forall i,j\\ \; & y_{ij}\in \left\{0,1\right\}, & \forall i,j\\ \; & x_{j}\in \left\{0,1\right\}, & \forall j \end{array}$$

where:

$b_{i}$: demand of *i*th customer

$d_{ij}$: distance between *i*th customer and *j*th candidate facility

*P*: number of facilities to be located

$x_{j}$: binary variable, =1 if locate facility at *j*th candidate, =0 if not

$y_{ij}$: binary variable, =1 if *i*th customer is served by *j*th facility, =0 if not

The optimal solution for $x_{j}$ and $y_{ij}$ indicates the locations of *P* facilities and the clients’ allocation that provides the minimum total distance between the clients and the facilities. As mentioned above, the goal of the P-median problem is to choose *P* locations from all candidates that optimize an objective function. It is analogous to the FRS problem, in which the goal is to locate a subset of features that maximize the mutual information between the selected features and the response variable. They both attempt to choose entities from a pool of all potential entities to achieve a specific goal. Because of this similarity, we apply MILP to the FRS problem and propose a new approach to select features by solving MI-transfer-network-based integer linear programming (MITN-ILP). Section 3.2 provides more details about this method.

### 3.2. The Proposed Algorithm (MITN-ILP)

The P-median problem and its similarities to the FRS problem were presented in the previous section. It has clearly demonstrated the feasibility of handling the feature selection problem by reframing MILP models for the P-median problem. Next, we give more details about the proposed MITN-ILP method, which includes two main steps listed below:

- First, create the MI transfer network from original data.
- Then, build the MILP model and solve it.

The solution of the MILP model designates the selection of features. In subsequent subsections, we present each step explicitly.

#### 3.2.1. Create the MI Transfer Network

**Figure 3:**
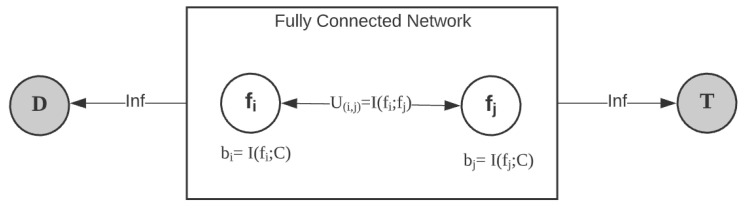
Structure of MI-transfer-network.

Before developing the MILP model for the FRS problem, we need to transform the information from the FRS problem into sets of variables and constraints for the MILP model, by constructing an MI transfer network. Figure 3 depicts the ubiquitous structure of MI transfer network.

The network within the square box is a fully connected network consisting of all candidate features, where the *i*th feature is represented by a node $f_{i}$ with a supply $b_{i}=I\left(f_{i};C\right)$, and the capacity of the arc between $f_{i}$ and $f_{j}$ is the MI between the *i*th feature and the *j*th feature. Furthermore, there is also a designated target node *T* for gathering all the transferred information, as well as a dummy node *D* for ensuring the network’s flow balance. The arc capacity from any feature node to node *T* or node *D* is infinite. After calculating the paired MI of the given data set, we can create the flow network as illustrated in Figure 3 and convert the FRS problem into an MILP problem that maximizes the flow into node *T*. In the MI-transfer-network, the communicated information between each paired predictors is quantitated by the MI between them, and the information of the response variable contained in each predictor is quantitated by the MI between it and the response variable. Since we assume that the communicated information between two predictors includes the joint information of the response variable contained in those two predictors, only one node of the fully connected network is permitted to transmit the MI into node T to restrict the redundancy of response information. Section 3.2.2 presents detailed MILP formulations for this problem.

#### 3.2.2. Develop the MILP Model

For the FRS problem, our aim is to select a set of features that maximize the MI between the selected features and the target variable. To clarify the formulation, we add some definitions before constructing the MILP model. First, an MI-Matrix called ***U*** is defined to represent the mutual information between each pair of nodes, as shown in Equation (10). $U_{n+1,f_{i}}$ and $U_{n+2,f_{i}}$ are equal to zero, while $U_{f_{i},n+1}$ and $U_{f_{i},n+2}$ are equal to *E* (an immense positive value that times zero is still zero to ensure the calculability) since nodes *D* and *T* only have incoming flows.

(10) $$U=\begin{array}{ccccccc} f_{1} & f_{2} & \cdots & f_{n} & D & T & \\ \left(\begin{array}{c} 0\\ I(f_{2};f_{1})\\ \vdots \\ I(f_{n};f_{1})\\ 0\\ 0 \end{array}\right. & \begin{array}{c} I(f_{1};f_{2})\\ 0\\ \vdots \\ I(f_{n};f_{2})\\ 0\\ 0 \end{array} & \begin{array}{c} \cdots \\ \cdots \\ \ddots \\ \cdots \\ \cdots \\ \cdots \end{array} & \begin{array}{c} I(f_{1};f_{n})\\ I(f_{2};f_{n})\\ \vdots \\ 0\\ 0\\ 0 \end{array} & \begin{array}{c} E\\ E\\ \vdots \\ E\\ 0\\ 0 \end{array} & \left.\begin{array}{c} E\\ E\\ \vdots \\ E\\ 0\\ 0 \end{array}\right) & \begin{array}{c} f_{1}\\ f_{2}\\ \vdots \\ f_{n}\\ D\\ T \end{array} \end{array}$$

Meanwhile, all mutual information between each feature and the target variable *C* is denoted by the supply vector ***b***:

(11) $$b=\left[\begin{array}{c} b_{1}=I\left(f_{1};C\right)\\ b_{2}=I\left(f_{2};C\right)\\ \vdots \\ b_{n}=I\left(f_{n};C\right) \end{array}\right]$$

The target variables for MILP are given by ***x***, ***y***, and ***v***, while both ***y*** and ***v*** are binary vectors.

(12) $$x=\left[\begin{array}{cccccc} x_{1,1} & x_{1,2} & \cdots & x_{1,n} & x_{1,d} & x_{1,t}\\ x_{2,1} & x_{2,2} & \cdots & x_{2,n} & x_{2,d} & x_{2,t}\\ \vdots & \vdots & \ddots & \vdots & \vdots & \vdots \\ x_{n,1} & x_{n,2} & \cdots & x_{n,n} & x_{n,d} & x_{n,t}\\ x_{d,1} & x_{d,2} & \cdots & x_{d,n} & x_{d,d} & x_{d,t}\\ x_{t,1} & x_{t,2} & \cdots & x_{t,n} & x_{t,d} & x_{t,t} \end{array}\right],\;y=\left[\begin{array}{c} y_{1}\\ y_{2}\\ \vdots \\ y_{n} \end{array}\right],\;v=\left[\begin{array}{c} v_{1}\\ v_{2}\\ \vdots \\ v_{n} \end{array}\right]$$

Let $x_{i,*}$ denote the *i*th row of a matrix ***x***, and $x_{*,j}$ denote the *j*th column of a matrix ***x***, where i,j∈{1,2,3,…,n,t,d}. Hence, the MILP model identifying *P* features among all potential candidates that transfer maximum MI in the network is given in Section 3.2.1 is:

(13) $$\begin{array}{ccc} \mathrm{maximize} & \sum x_{*,n+2} & \\ \mathrm{subject}\;\mathrm{to} & e^{T}y=P\\ \; & e^{T}v=1\\ \; & \sum x_{i,*}-\sum x_{*,i}=b_{i}, & \;\forall i\\ \; & x_{*,n+2}\leq U_{*,n+2}\circ v\\ \; & x_{i,n+2}\leq k, & \;\forall i\\ \; & x_{*,n+2}\leq U_{*,n+2} \end{array}$$

where:

***e***: a column vector $={\left(1,1,\ldots ,1\right)}^{T}$, and $e^{T}$ is its transpose

***U***: MI-Matrix, where ***U*** the arc capacity between node *i* and node *j*

***b***: supply vector, where $b_{i}$ = the amount of information at node *i*

***x***: flow matrix, where $x_{i,j}$ = the amount of information shifted from node *i* to node *j*

***y***: binary vector, where $y_{i}=1$ if $f_{i}$ is selected and $y_{i}=0$ if not

***v***: binary vector, where $v_{i}=1$ if the arc $\left(f_{i},T\right)$ is activated and $v_{i}=0$ if not

*P*: total number of features are selected

*k*: the precalculated parameter for a better bound for the solution

∘: the Hadamard product operator

**Figure 4:**
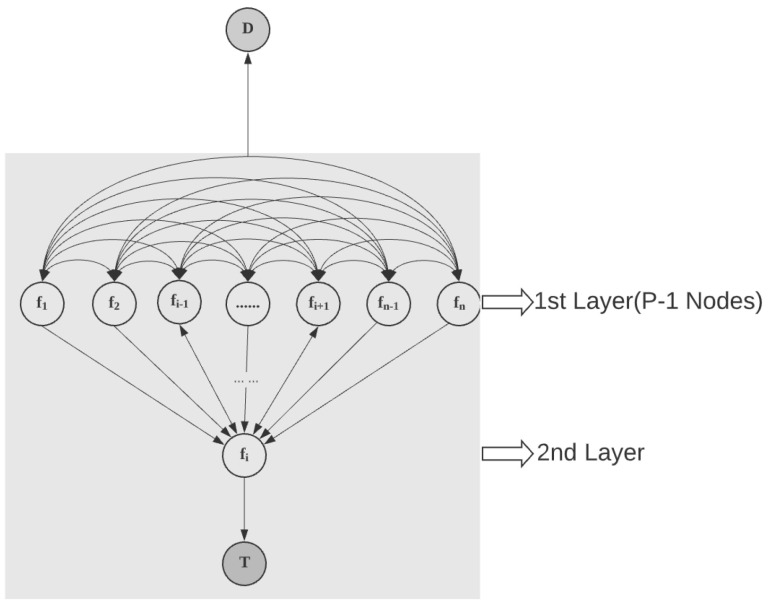
End part of MI-transfer-network.

It should be noted that in the proposed method, only one node can transmit the MI into node *T*, as indicated by $e^{T}v$ in the above formulation. Without this constraint, the selected nodes will be the top *P* nodes by their supply, which does not meet our expectations. Furthermore, an upper bound k is also applied to reduce the calculation time by shrinking the feasible area. To obtain an appropriate *k*, we separate all the nodes into two types based on their value of ***v***. All nodes with $v_{i}=0$ form the first layer, while the nodes with $v_{i}=1$ constitute the second layer as displayed in Figure 4.

If the *i*th node is selected from the second layer, then only the (P−1) nodes can be selected from UNS, where UNS is a set composed of all the unselected features, to form the first layer. The maximum transferable MI from the first layer into the second layer should be less than the maximum total supplies in the first layer, which is the sum of the top (P−1) values in $\left\{b_{j},\forall j\in \mathrm{UNS}\right\}$. Moreover, the maximum MI should also be limited by the maximum total capacities between the first layer and the second layer, which is the sum of the top (P−1) values in $\left\{U_{j,i},\forall j\in \mathrm{UNS}\right\}$. Consequently, we can obtain one upper limit *k* (the right side of the inequality given below) for the total maximum flow:

(14) $$x_{i,n+2}\leq \min\left\{b_{i}+\sum max_{P-1}\{U_{j,i},\forall j\in \mathrm{UNS}\right\},b_{i}+\sum max_{P-1}\{b_{j},\forall j\in \mathrm{UNS}\}\}$$

To compute optimal solutions, various solvers may be utilized. We used the Gurobi solver in this study. As stated in Section 3.1, it is not easy to find the global optimum efficiently, especially for the high-dimensional data. Therefore, the relative MIP optimality gap (MIPGap) is adopted as the termination criterion for the Gurobi solver [35]. MIPGap refers to at least the gap value that Gurobi has to reach before declaring optimality, and a larger MIPGap usually reduce the computational time. Optimal values of ***x***, ***y***, and ***v*** under this criterion are obtained from the solver. The optimal value of ***y*** is the most essential result, since it indicates the selection of the features.

## 4. Experiments and Results

To verify the performance of our proposed algorithm, we applied the algorithm in conjunction with six other FRS methods: mRMR, CMIM, CIFE, Relief [36], CFS [37], and Fisher’s Score (FS), to a set of benchmark data sets, then compared their classification accuracy. Unlike the other six methods, MITN-ILP requires a mathematical optimization solver. We applied AMPL [38] as the algebraic modeling language cooperating with Gurobi, and increased MIPGap from its default value 0.01% to 5% which reduced the computational time by 88.3%. The summary of the data sets is given in Table 2, which contains the number of observations, features, and classes. Data sets comprise of both binary data sets and multiclass data sets from various aspects. The data sets are from the UCI machine learning repository [39], except Mfeat and Volkert that are from OpenML [40].

To build classification models, all seven FRS methods, including our proposed method, have been used to select the features with feature numbers P={5,10,15,…,50}. Afterward, there are four kinds of model: K-Nearest- Neighbor (KNN) [41], Naïve Bayes (NB) [42], Support Vector Machine (SVM) [43], and Linear Discriminant Analysis (LDA) [44], are implemented to exclude the influences of classifiers. Finally, the overall performance of each FRS method is measured by the average classification accuracy obtained from these four models.

**Figure 5:**
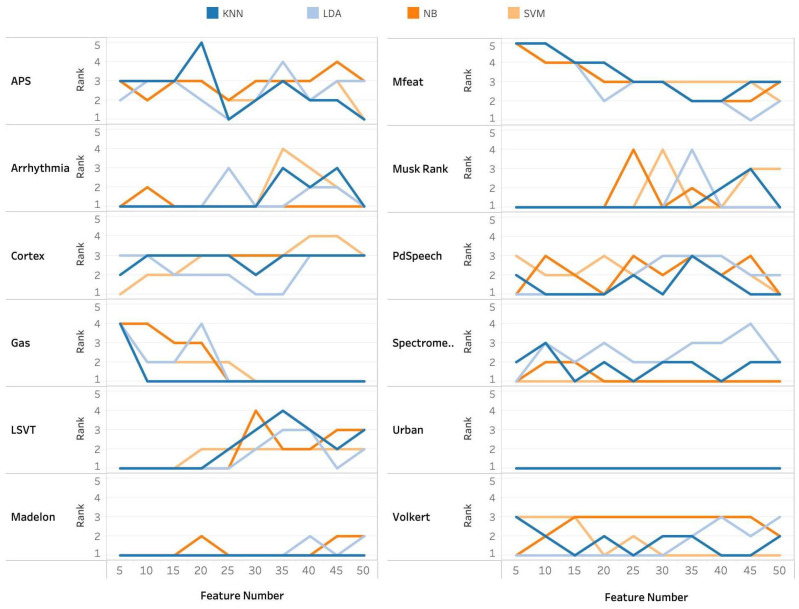
Classification accuracy rank of MITN-ILP for each data and classifier.

**Figure 6:**
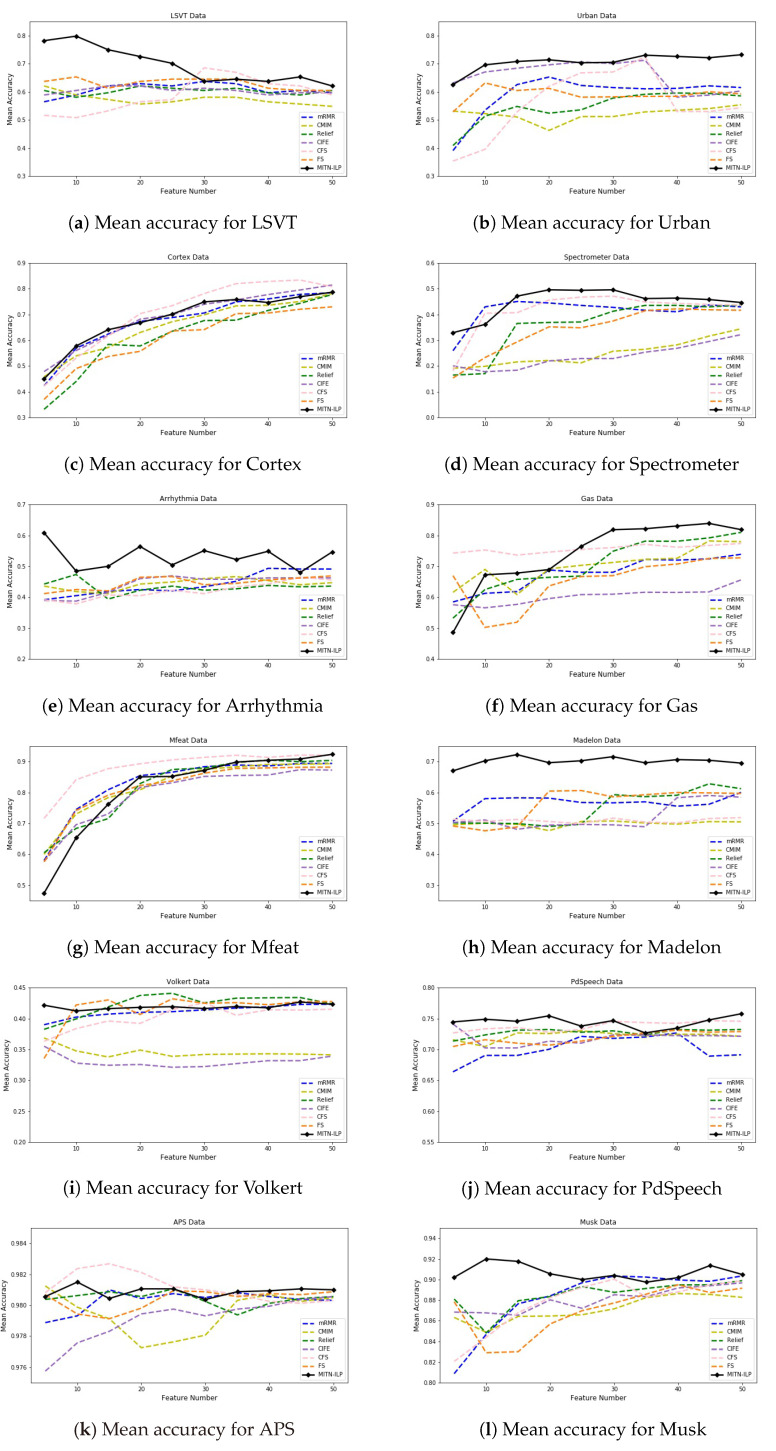
Mean classification accuracy for all our data.

Experimental results are presented in Figure 5 and Figure 6. The classification accuracy of FRS methods with each classifier has been ranked in descending order. The rankings of the proposed approaches for each data and classifier are shown in Figure 5. Moreover, Figure provides 12 subfigures reporting the average classification accuracy from all classifiers of 12 data sets. Different colors denote different FRS methods, and the solid black line symbolizes our proposed method. The proposed method outperforms all other methods in terms of mean accuracy across almost all other data sets, and it ranks among the top three overall when the number of selected features is greater than 20.

**Figure 7:**
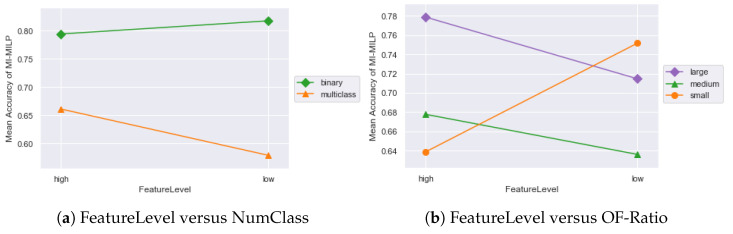
Interaction plots for mean accuracy.

Despite the fact that our proposed method shows its superiority, there is some slight variation across data sets. For instance, MITN-ILP’s superiority is significant when p≤ 25 for the Musk and LVST, but it is not for the Gas and Mfeat. To figure out the factors that might account for the discrepancy of the proposed method, analysis of variance (ANOVA) is used to detect significant factors from: the number of selected features (FeatureLevel), the number of classes (NumClass), and OF-Ratio= $\frac{\left|observations\right|}{\left|features\right|}$. OF-Ratio is arisen here instead of directly using the number of features and the number of observations, because it standardized the number of observations and the number of features ranging from hundreds to billions in real-world problems. Before further analysis, we separate OF-Ratio into three levels: (0, 1] for ‘small’, (1, 10] for ‘medium’, and (10, *∞*) for ‘large’. The number of classes is separated into ‘binary’ and ‘multiclass’, while the number of selected features is categorized into [5, 25] (‘low’) and [30, 50] (‘high’). The interaction plots from ANOVA are given in Figure 7.

The *p*-value of interaction effect between FeatureLevel and NumClass is 0.029, while that between FeatureLevel and OF-Ratio is 0.046. Therefore, we can conclude the significance of these interaction effects. From the interaction plots, the MITN-ILP performs better when FeatureLevel = ‘low’ for binary classification and FeatureLevel = ‘high’ for multiclass. Furthermore, MITN-ILP provides higher accuracy for the data with an OF-Ratio > 10 when selecting more than 25 features. For the data set with an OF-Ratio ≤ 1, MITN-ILP performs better when choosing a smaller size of features.

## 5. Discussion and Conclusions

In this study, we proposed a new approach that attempts to overcome the shortcomings of MIGFMs, such as the ignorance of relatively optimum feature subsets, the sensitivity to parameters, and the stopping criterion. By converting it into a maximum flow problem in the MI transfer network, we are able to solve it without considering the necessary parameters for MIGFM. As mentioned in Section 3, MILP can be time-consuming when it involves too many variables and constraints. Therefore, we introduce an upper limit to MI-transfer-network-based linear programming model for computational time reduction.

To analyze the performance of the proposed method, we designed experiments to select features for classification problems with 12 data sets in our proposed method and the other 6 FRS methods. The results achieved from these comparative experiments demonstrated MITN-ILP’s superiority in feature selection concerning classification precision, especially for high-dimensional data that have fewer observations. The insights from this study can assist the healthcare or clinical investigation that are more sensitive to precision and suffers from a lack of patient records, such as electronic health records (EHRs) [45] for newly established healthcare or rural hospitals. Furthermore, unlike greedy forward FRS methods, MITN-ILP helps avoid potential negligence of a better predictor set, since it is unaffected by the preset parameters and the current greedy state.

Despite these encouraging results, questions remain since the complexity of the converted MILP problem increases with the size of the data sets, particularly in terms of the number of features. While our proposed method achieves superior performance efficiently with the aforementioned upper limit of maximum flow and the optimality gap, future work should consider whether a better upper limit can be generalized to make our approach feasible for extremely large data. Furthermore, an acceptable optimality gap that balances efficiency and selection performance is also essential, which allows a broader range of applications of our proposed method.

## Figures and Tables

**Table 1:** MI-based FRS methods in summarized algorithm format.

MI-Based FRS Methods	β	$g(f,f_{i},C)$
MIFS-U	β	$\frac{I(f_{i};C)}{H(f_{i})}I\left(f;f_{i}\right)$
IGFS	−$\frac{1}{\left|SEL\right|}$	$I(f;C,f_{i})$
CIFE	1	$I(f_{i};C,f)$
MRMD	$\frac{1}{\left|SEL\right|}$	$I\left(f;f_{i}\right)-I\left(f;C|f_{i}\right)$
MRI	1	$2I\left(f;f_{i};C\right)-I\left(f;C\right)-I\left(f_{i};C\right)$
mRMR	$\frac{1}{\left|SEL\right|}$	$I(f;f_{i})$
SPEC-CMI	−1	$I(f;C|f_{i})$

**Table 2:** Summary of data sets.

Data Sets	Number of Observations	Number of Features	Number of Classes
APS	60,000	170	2
Arrhythmia	279	179	16
Cortex	1080	81	8
Gas	13,910	128	6
LSVT	126	310	2
Madelon	4400	500	2
Mfeat	2000	217	10
Musk	6598	168	2
PdSpeech	756	754	2
Spectrometer	531	102	48
Urban	675	148	9
Volkert	58,310	181	10

## Data Availability

Not applicable.
